# Modeling of the Production of Lipid Microparticles Using PGSS^®^ Technique

**DOI:** 10.3390/molecules25214927

**Published:** 2020-10-24

**Authors:** Clara López-Iglesias, Enriqueta R. López, Josefa Fernández, Mariana Landin, Carlos A. García-González

**Affiliations:** 1 Department of Pharmacology, Pharmacy and Pharmaceutical Technology, I+D Farma group (GI-1645), Faculty of Pharmacy, Agrupación Estratégica de Materiales (AeMAT) and Health Research Institute of Santiago de Compostela (IDIS), Universidade de Santiago de Compostela, 15782 Santiago de Compostela, Spain; m.landin@usc.es; 2 Laboratorio de Propiedades Termofísicas, Grupo NaFoMat, Departamento de Física Aplicada, Facultad de Física, Agrupación Estratégica de Materiales (AeMAT), Universidade de Santiago de Compostela, 15782 Santiago de Compostela, Spain; enriqueta.lopez@usc.es (E.R.L.); josefa.fernandez@usc.es (J.F.)

**Keywords:** lipid microparticles, PGSS^®^, supercritical CO_2_, modeling, solvent-free technology

## Abstract

Solid lipid microparticles (SLMPs) are attractive carriers as delivery systems as they are stable, easy to manufacture and can provide controlled release of bioactive agents and increase their efficacy and/or safety. Particles from Gas-Saturated Solutions (PGSS^®^) technique is a solvent-free technology to produce SLMPs, which involves the use of supercritical CO_2_ (scCO_2_) at mild pressures and temperatures for the melting of lipids and atomization into particles. The determination of the key processing variables is crucial in PGSS^®^ technique to obtain reliable and reproducible microparticles, therefore the modelling of SLMPs production process and variables control are of great interest to obtain quality therapeutic systems. In this work, the melting point depression of a commercial lipid (glyceryl monostearate, GMS) under compressed CO_2_ was studied using view cell experiments. Based on an unconstrained D-optimal design for three variables (nozzle diameter, temperature and pressure), SLMPs were produced using the PGSS^®^ technique. The yield of production was registered and the particles characterized in terms of particle size distribution. Variable modeling was carried out using artificial neural networks and fuzzy logic integrated into neurofuzzy software. Modeling results highlight the main effect of temperature to tune the mean diameter SLMPs, whereas the pressure-nozzle diameter interaction is the main responsible in the SLMPs size distribution and in the PGSS^®^ production yield.

## 1. Introduction

Particulate systems like microparticles have attracted interest in several biomedical, food and environmental applications [1,2,3,4,5]. Namely, the encapsulation of bioactive agents in these carriers improves their efficacy and safety, since better control of the dosage and release are provided [6,7]. Microparticles also enhance physicochemical stability, protecting the cargo from environmental and physiological factors [8]. The size of microparticles, between 0.1–100 μm [9], can hamper their absorption through biological membranes, increasing their permanence in the application site, thus providing local and sustained drug release and mitigating their toxic effects [10].

Lipids are advantageous matrices for particulate drug delivery systems since they are physiological compounds and therefore well tolerated by living systems [11,12]. For instance, a variety of lipids such as sorbitan esters, phosphatidylcholine, and unsaturated polyglycolized glycerides are widely used as surfactants in lipid-based formulations [13]. Among lipid systems, solid lipid microparticles (SLMPs) are easy to produce on a large scale and sterilize, exhibiting better stability properties than others, such as liposomes [14]. Several SLMP-based formulations have been developed as drug delivery systems for oral, parenteral, pulmonary and topical applications [14,15].

Solvent-free strategies are especially attractive for the manufacturing of SLMPs from the processing, environmental and economical points of view. Namely, supercritical CO_2_ (scCO_2_) technology has been highlighted as a processing tool for environmentally friendly, safe and cost-efficient techniques at mild conditions—pressure (P) > 73.8 bar and temperature (T) > 31.1 °C) [16]. Processes based on supercritical fluid technology (foaming, sterilization) usually avoid or at least mitigate the use of organic solvents thus reducing their carbon footprint. The PGSS^®^ (Particles from Gas-Saturated Solutions) technique is based on the use of compressed CO_2_ or scCO_2_ for the production of microparticles in an atomization-wise process [17,18,19]. PGSS^®^ process comprises two main steps: (i) CO_2_ sorption in the polymer, and (ii) polymer expansion and particle formation. In the first step, high amounts (5–50 wt.%) of CO_2_ dissolve in a molten substance at a moderate pressure in an extent depending on the soaking time and CO_2_ affinity to the polymer [20]. Then a rapid expansion to atmospheric pressure of the melt through a nozzle causes an intense cooling effect and CO_2_ supersaturation within the melt, resulting in the precipitation of solid particles [21]. scCO_2_ used in the PGSS^®^ technique differs from other compressed fluids (e.g., compressed air) used in conventional atomization processes (spray drying) in their chemical interaction with the processed polymers at a molecular level, as scCO_2_ can decrease the melting temperature of the polymer thus contributing to costs optimization and energy consumption savings [22]. PGSS^®^ is an adequate technique for the processing of polymeric particles incorporating thermolabile compounds, although its use is limited to polymer matrices with relatively low melting temperatures and with an affinity of CO_2_ to the polymer [23]. Compared to other processes for particle production involving the use of scCO_2_, such as the gas antisolvent (GAS), supercritical antisolvent (SAS) and supercritical fluid extraction of an emulsion (SFEE) techniques, the PGSS^®^ technique does not use any organic solvents [16,24]. Moreover, the substance to be micronized does not require to be soluble in CO_2_ unlike in the rapid expansion of supercritical fluids (RESS) process [25,26]. Overall, PGSS^®^ emerges as an appealing and advantageous technique for the processing of SLMPs at reduced melting temperatures and in the absence of organic solvents.

The morphology and size of the SLMPs produced by the PGSS^®^ process are mainly influenced by the formulation (chemical composition and rheology of the compounds to be precipitated), the technical details of the equipment used (volume of the saturator, precipitator and collector, diameter of the nozzle and length of the tubing) and the operating conditions (pressure, temperature, soaking time) [27,28]. The PGSS^®^ processing variables are numerous, making it difficult to elucidate their influence on the characteristics of the microparticles using conventional statistical methods [29,30,31]. Despite PGSS^®^ being a simple and versatile method, the lack of knowledge of the effects of the variables on the results of PGSS^®^ technology may entail an obstacle towards the robust SLMPs production and the scaling-up of the process [32]. Approaches based on DoE (design of experiments) and multiple regression have been proposed to manage the number of experiments, to select the critical variables and to optimize the operation conditions, but mainly regarding their influence on the dissolution profile of the drug incorporated in the particles [33]. Some mathematical models were also proposed to simulate the physicochemical processes taking place during the PGSS^®^ processing, such as the behavior of a CO_2_-supersaturated solution drop in low-pressure environments [34,35]. In this context, artificial intelligence technologies emerge as tools with great potential for simplifying the study of processes in which many variables are involved, even when a small number of experiments are available. Some of them, such as the neurofuzzylogic systems, allow multiple variables to be modeled and the models expressed through language, which generates in-depth knowledge about the process. Neurofuzzylogic software is a hybrid system that combines artificial neural networks (ANN) and fuzzy logic (FL). ANN are computer programs that simulate how the human brain processes information. They detect patterns and relationship in data, and learn from experience, leading to “black-box” mathematical models [36]. When combined with FL, the models are expressed as simple linguistic IF…THEN rules together with a membership degree, losing their black-box character and being easily understandable.

Artificial intelligence tools have been previously used in the development and optimization of microparticles [37] and polymeric and lipid nanoparticles [38,39]. To the best of our knowledge, these tools are applied in this work for the first time to model the production of SLMPs by the PGSS^®^ technology. SLMPs consist of a matrix of commercial glyceryl monostearate (GMS), a lipid widely used as an emulsifier in pharmaceutical preparations due to its good biocompatibility and safety [40,41]. First, the melting point depression of commercial GMS in contact with scCO_2_ was studied to establish the limits of the adequate knowledge space for the processing of PGSS^®^. Subsequently, an unconstrained D-optimal design for three variables (nozzle diameter, pressure and temperature) at 2, 3 and 3 levels, respectively, was used to prepare SLMPs using the PGSS^®^ technique. The microparticles were characterized in terms of size and shape. The generated database was modeled through a neurofuzzylogic system and the design space was established with respect to the melt GMS processability (fine particle production yield) and the characteristics of the particles.

## 2. Results and Discussion

### 2.1. Melting Point Depression of GMS in the Presence of CO_2_

**Figure 1:**
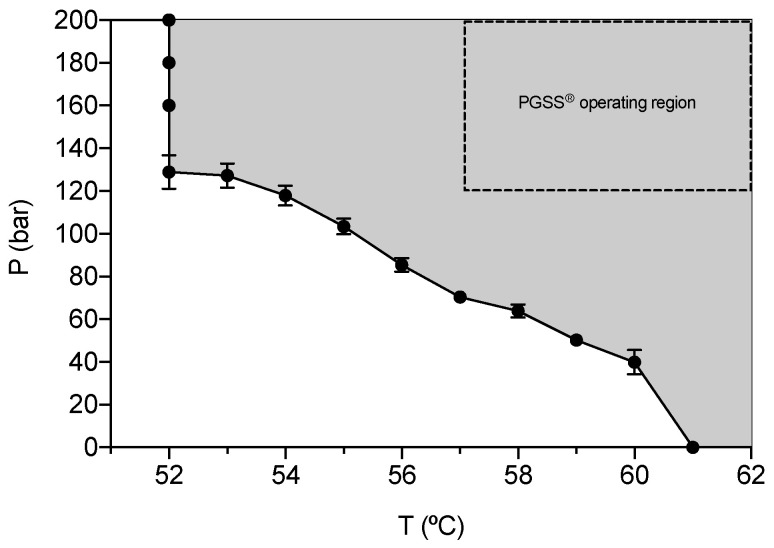
Glyceryl monostearate (GMS) melting points obtained at different pressures of CO_2_ using a variable-volume high-pressure view cell. Grey area represents the pressure-temperature region at which GMS will be molten. The area delimited by the dashed line represents the operating region established for solid lipid microparticles (SLMPs) production by PGSS^®^ technique.

Melting pressure-temperature curve of the commercial GMS under compressed CO_2_ was measured to determine the feasible operating range of conditions for the PGSS^®^ technique (Figure 1). This step is crucial since it is necessary to establish a set of pressure-temperature conditions (grey region in Figure 1) where the lipid mixture is molten. The melting point of GMS in the presence of CO_2_ has been previously studied [42], but these determinations are essential because it is well known that GMS can have inter-batch and inter-manufacturer variability as it is commercially provided as a mixture of components (mono- and diglycerides).

The melting point of the commercial GMS without CO_2_ was 61 °C at ambient pressure. CO_2_ can act as a plasticizer agent, being able to melt other substances, like lipids or polymers, below their normal melting points. Melting point depletion effect of GMS in contact with CO_2_ is highly dependent on the working pressure and decreased proportionally up to 52 °C as can be seen in Figure 1. This effect was related to the increase in the amount of CO_2_ dissolved in the lipid when the pressure increases [43]. A plateau in temperature was reached at 52 °C and pressures above 120 bar were not able to cause an additional melting point depletion. This second effect was related to the competing mechanism of increased CO_2_ solubility in the lipid and the hydrostatic pressure promoting the melting point depletion and increase, respectively, that are counteracting at pressures above 120 bar for GMS [42]. The reduced melting temperature in the presence of compressed CO_2_ is advantageous for the energy optimization of the PGSS^®^ particle processing when transferring formulations from lab to pilot scale [44,45].

### 2.2. Particle Size Distribution (PSD), Morphological and Physichochemical Characterization of GMS Particles

Based on the melting point values obtained in Section 2.1, the range of values of pressure and temperature selected for the experimental study of the PGSS^®^ processing of GMS particles were set at 120–200 bar and 57–67 °C, respectively. In this work, an increment of ca. 5 °C with respect to the melting temperature of GMS at a certain pressure in the presence of compressed CO_2_ was established as a rule-of-thumb (dashed and grey rectangle in Figure 1) to ensure the complete melting and to avoid clogging of the nozzle during the PGSS^®^ expansion-spraying step. The selection of the nozzle diameter was based on the technical possibilities of the PGSS^®^ equipment, being 4 and 1 mm the maximum nozzle diameter and the minimum nozzle diameter that did not cause clogging events upon depressurization using the established P-T range in the experimental design, respectively.

**Figure 2:**
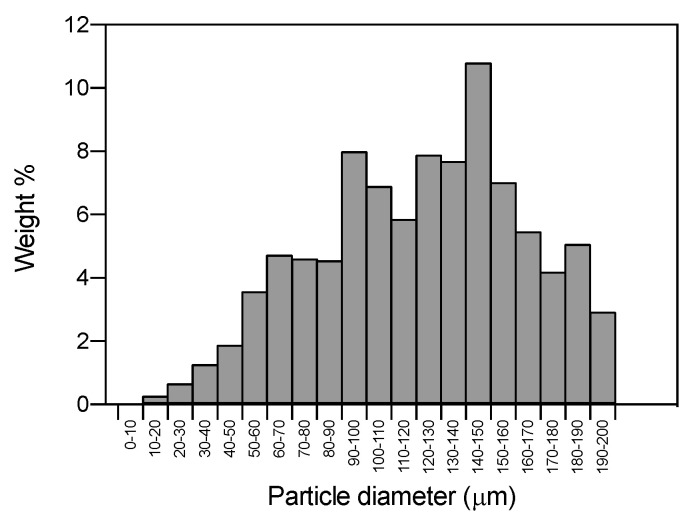
Frequency histogram of GMS-1-67-200 particles (mean particle diameter = 125.4 ± 43.1 μm). The normal distribution of this histogram is representative of all the GMS formulations tested.

PSDs of the SLMPs showed mean diameters between 100 and 190 μm and standard deviations between 30 and 65 μm (Table 1). In general, the PSDs fitted well to a normal distribution (Figure 2) with good correlation levels (R^2^ > 0.95) in all cases. The yield of particle production was determined from the weight percentage of fine particles with respect to the initial GMS (Table 1). The loss of material during the PGSS^®^ processing was due to GMS remaining in the tubing and the saturator of the equipment, molten material that was not solidified into particles and formed a crust in the walls of the precipitator. Some mass losses were attributed to small particles that remained suspended in the outlet gaseous stream and were vented out during the depressurization step along with the CO_2_.

**Figure 3:**
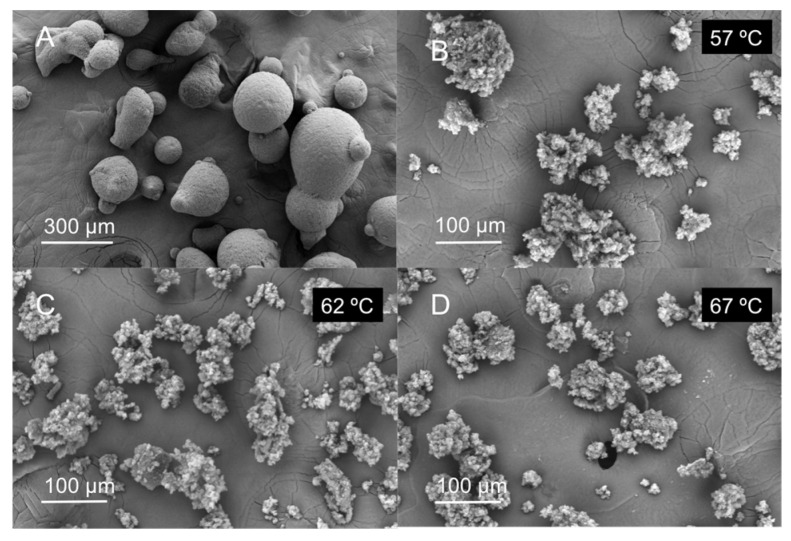
Effect of temperature in the PGSS^®^ processing of GMS particles: (**A**) unprocessed GMS particles and (**B**) GMS-1-57-200, (**C**) GMS-1-62-200 and (**D**) GMS-1-67-200 particles.

**Figure A1:**
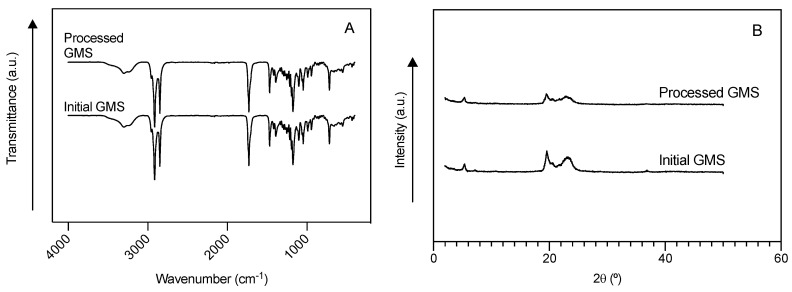
(**A**) ATR/FT-IR spectra and (**B**) XRD patterns of raw GMS particles and GMS particle processed by PGSS^®^.

The processing using PGSS^®^ technique led to particles with reduced circularity (60.7 ± 18.2%) with respect to the original GMS (round particles, Figure 3A). PGSS^®^-processed lipid microparticles had a decreased bulk density (0.14 g/cm^3^) with respect to the raw material (0.53 g/cm^3^). However, skeletal density was similar (0.995 ± 0.017 g/cm^3^) to the unprocessed GMS (0.980 ± 0.003 g/cm^3^), suggesting that the chemical structure of the GMS was not unaltered during the process, as also confirmed by X-ray diffraction (XRD) and Attenuated Total Reflectance/Fourier Transform infrared spectroscopy (ATR/FT-IR) (Figure A1).

### 2.3. Morphological Characterization and Modeling of GMS Particle Production Using Neurofuzzy Tool

**Figure 4:**
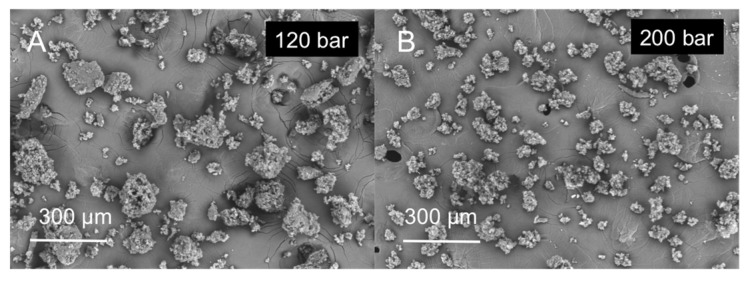
Effect of pressure in the PGSS^®^ processing of GMS particles: (**A**) GMS-1-67-120 and (**B**) GMS-1-67-200 particles.

The processing of GMS using the PGSS^®^ technique resulted in porous particles of varied shape and of lower particle diameter than the original material (Figure 3 and Figure 4).

Neurofuzzylogic software succeeded in modeling the influence of the parameters of pressure, temperature and nozzle diameter (inputs) on the output mean diameter (Table 2) with high predictability (R^2^ > 90%) and accuracy (*p* < 0.01). The three parameters help to explain the variations in particle size, with temperature (submodel 1) having the main effect. An interaction between the pressure and the nozzle can be also observed (submodel 2).

The predictability is also reasonable for the percentage of fine particles (R^2^ > 75%), a parameter indicative of process yield (Table 2). However, adequate accuracy was not achieved with such a small number of degrees of freedom. The model shows a main effect for the interaction pressure-nozzle, but temperature also affects process yield.

Variables studied do not explain sufficiently the variations in the standard deviation of the particle size distribution (R^2^ < 75%). The particle size distributions with PGSS^®^ technique are broad and characterized by high standard deviations, probably higher than the variations promoted by the processing parameters (temperature, pressure and nozzle diameter) used in this research. Therefore, the ANN cannot define a good model for this standard deviation.

IF…THEN rules, generated by the neurofuzzylogic software allows acquiring knowledge in an easy way (Figure A1). According to these rules, IF the temperature is low (up to 62 °C) THEN the mean particle size obtained is high (over 144.8 µm). The increase in temperature over 62 °C produces a decrease in particle size (Figure 3).

**Figure 5:**
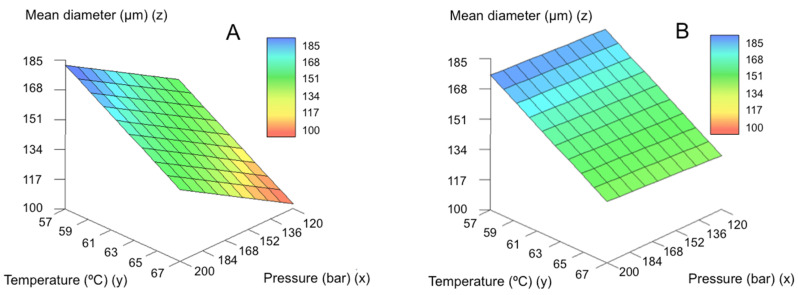
Predicted results by the model for mean particle size for the (**A**) large and (**B**) small nozzles.

On the other hand, (IF) the pressure increase (…THEN) promotes a decrease in the particle size of the microparticles (Figure 4). This rule applies for both small and large nozzle diameters, being the variations in particle size wider when the large nozzle is used. Figure 5 represents the predicted results by the model for mean particle size for the large (Figure 5A) and small (Figure 5B) nozzle. This effect was related to the increased solubility of CO_2_ in molten GMS. At higher pressures CO_2_ solubility will increase and, upon depressurization, more nucleation bubbles will form due to CO_2_ supersaturation, breaking the lipid into smaller particles (Figure 4) [42,46]. Using the large nozzle diameter, pressure variations produced a more pronounced effect on the mean particle diameter.

**Figure 6:**
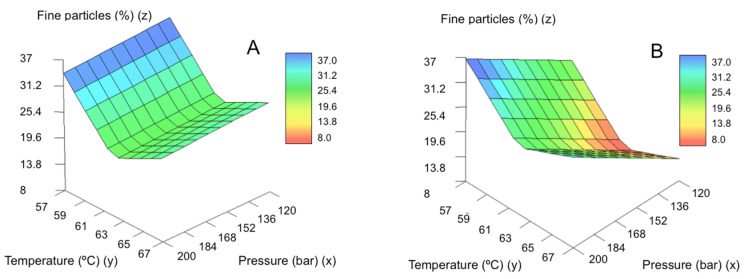
Influence of the parameters pressure and temperature on the yield of fine particle formation using: (**A**) the large nozzle diameter and (**B**) the smaller nozzle diameter.

Figure 6 shows the predicted values for the percentage of fine particles as a function of pressure and temperature. The increase in temperature leads to a reduction in the process yield, being especially important up to 62 °C. In the temperature range of the experimental design (57–67 °C), the Joule-Thomson coefficient is very similar for the 0–200 bar pressure range [47]. At higher temperatures, the positive Joule-Thomson effect contribution may not be enough to solidify the GMS when exiting the nozzle. Under these conditions, a significant fraction of GMS is in a semi-molten state when it reaches the precipitator and forms a crust in the walls of the vessel instead of forming SLMPs that deposit on the collector.

The diameter of the nozzle also influenced the fine particle yield production. In general, the process performs better when using the small size nozzle. It has been reported that lower nozzle diameters led to smaller particle sizes for other lipid-based systems [48]. Differences in the effect of pressure were also detected depending on the size of the nozzle used. When a nozzle of smaller size is used, the increase in pressure causes a slight reduction in the percentage of fines obtained. This may be related to the production of even smaller particles that remain suspended in the CO_2_ and are therefore vented out. However, when the nozzle has a larger diameter, the effect is the opposite, and the process performance is improved with increasing pressure. The pressure drop of the lipid-CO_2_ melt through the nozzle is lower with larger nozzle diameters, leading to a decreased Joule-Thomson cooling effect. At higher pressures, this pressure drop effect is compensated by a higher CO_2_ content in the lipid melt and particles are able to solidify and reach the collector leading to higher fine particle yield [49].

**Figure 7:**
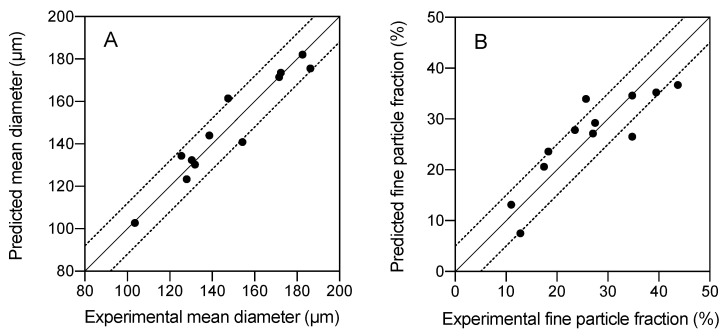
Parity plots of the predicted and experimental values of (**A**) mean particle size and (**B**) % of fine particles. Continuous diagonal line is a 45°-slope line; dotted lines correspond to an envelope of tolerance of 10%.

The experimental values were compared with the values predicted with the model, showing high accuracy of the models for fine particle fraction (Figure 7A) and mean diameter (Figure 7B).

## 3. Materials and Methods

### 3.1. Materials

Kolliwax^®^ GMS II (glycerylmonostearate 40–55 type II, powder, Tm = 54–64 °C) was supplied by BASF GmbH (Ludwigshafen am Rhein, Germany). CO_2_ for the PGSS^®^ technique (purity 99.8%) and for the melting point determination (purity 99.998%) were purchased from Praxair (Madrid, Spain) and Air Liquide (Santiago de Compostela, Spain), respectively.

### 3.2. Determination of the Melting Point of GMS in the Presence of Compressed CO_2_ at Different Pressures

The melting point of the GMS in the presence of compressed CO_2_ in a 0–200 bar pressure range was determined. A sample of GMS (approximately 3.5 mg) on a glass vial was placed inside a variable volume high-pressure cell, consisting of a horizontal stainless-steel cylinder with an internal diameter of 2 cm and a piston to adjust the volume from 7.9 to 29.5 cm^3^. The cell was equipped with a sapphire window (1.6 cm diameter) that allowed the detection of phase transitions through an endoscope (Olympus 5 series, Olympus, Tokyo, Japan) connected to a CCD-camera (Moticam 2000, Motic Asia, Hong Kong, China). In one sidewall of the cylinder, a second sapphire window (6 mm in diameter) made it possible to illuminate the interior of the cell through an optical fiber. A Pt100 probe with an uncertainty of 0.02 °C was used to measure the temperature in the cell wall. The pressure was measured with a Heise model DXD series digital pressure transducer, with an operating range 0–500 bar and an uncertainty of 0.02% of the full scale (FS).

For the experimental trials, the cell at its maximum volume was filled with CO_2_ at room temperature and supply pressure of 60–65 bar. Afterwards, the system was heated to the selected temperature (from 52 to 61 °C) and the pressure was gradually increased moving the piston (i.e., reducing the volume of the chamber) until the solid was completely molten to determine the melting point value. Thus, the melting pressure of the GMS at the selected temperature was determined. Subsequently, another temperature was selected and the procedure was repeated to obtain another value of the melting curve. Temperature measurements were carried out by triplicate. Results were expressed as the mean value ± standard deviation (SD). At a fixed temperature, this device shows repeatability for the pressure lower than 11.4%. The melting point temperature of the GMS at atmospheric pressure in the same equipment was also determined.

### 3.3. SLMPs Production by the PGSS Technique

For the particle formation protocol, 6 g of GMS powder were placed into a 250-mL high-pressure autoclave (saturator) (Eurotechnica GmbH, Bargteheide, Germany). After heating the saturator to the desired temperature (T), CO_2_ entered the equipment at a constant flow of 7 g/min until the desired pressure (P) was reached. After 1 h of contact between the molten lipid and the compressed CO_2_ under stirring at 400 rpm, the system was depressurized by opening the valve placed at the bottom of the saturator. When the molten lipid leaves the saturator through a nozzle, rapid depressurization causes lipid microparticles precipitation within a 2.7 L borosilicate autoclave (precipitator).

Batches of GMS particles were produced following a D-optimal experimental design for three variables: nozzle diameter (2 levels), operating temperature (3 levels) and pressure (3 levels) (Table 3) carried out by DataForm^®^ v.3.1 software (Intelligensys Ltd., Stokesley, UK). GMS particles processed under different pressure and temperature conditions were denoted as GMS-x-y-z, where x is the nozzle diameter in mm, y the processing temperature in degrees Celsius and z the processing pressure in bar.

Microparticles were collected and weighed to determine the process yield according to Equation (1):

(1) $$\%\;\mathrm{fine}\;\mathrm{particles}\;=\;\frac{W_{f}}{W_{0}}\;\times \;100$$

where W_0_ is the initial weight of GMS added to the saturator and W_f_ is the final weight of fine particles collected. Also, the amount of GMS remaining on the walls of the precipitator and the interior of the tubing was weighed to verify all the GMS had left the saturator, and what amount had not precipitated into SLMPs.

### 3.4. Morphological Analysis, Physicochemical Characterization and Particle Size Distribution (PSD)

Four aliquots of each batch were characterized in terms of particle size distribution by optical microscopy using a camera (EP50, Olympus, Tokyo, Japan) provided with the software EP View (Olympus, Tokyo, Japan). The images were analyzed using the freeware ImageJ 1.49v. Calculated particle diameters correspond to the projected area equivalent diameter. The particle size distributions were fitted to a normal distribution, and mean particle size and standard deviations were obtained. The circularity of the particles was also evaluated by image analysis.

X-ray diffraction (XRD) and attenuated total reflectance/fourier transform infrared spectroscopy were used to test possible physicochemical modifications in GMS caused by PGSS^®^ processing. XRD patterns were collected (PW-1710, Philips, Eindhoven, The Netherlands) in the 2–50° 2θ-range using a 0.02° step and CuKα_1_ radiation. ATR/FT-IR spectra (Gladi-ATR, Pike, Madison, WI, USA) were obtained in the 400–4000 cm^−1^ spectrum range from 32 scans and at a resolution of 2 cm^−1^.

Particles were also analyzed by scanning electron microscopy (SEM Zeiss EVO LS 15; Zeiss, Oberkochen, Germany) to evaluate their morphology and surface texture. Particles were previously sputtered-coated with a layer of 10 nm of iridium to improve the contrast (Q150 T S/E/ES, Quorum Technologies, Lewes, UK). Bulk density of the particles was determined by a volumetric method and the skeletal density was evaluated using helium pycnometry (MPY-2; Quantachrome, Delray Beach, FL, USA).

### 3.5. Modeling

The generated database (inputs from Table 3 and outputs from Table 1) was modeled using the commercial software FormRules^®^ v4.03 (Intelligensys Ltd., Stokesley, UK) which is a hybrid system that combines Artificial Neural Networks (ANN) and fuzzy logic. Nozzle diameter, pressure and temperature were introduced as inputs, while percentage of fine particles, mean particle size and standard deviation were introduced as outputs. A separate model was developed for each output. These models are split into different submodels, when it is possible, to generate simple and understandable rules.

Among the fitness criteria included by FormRules^®^ (cross validation, minimum description length, structural risk minimization, leave one out cross validation and Bayesian information criterion), minimum description length was selected because it gives the best R-squared as well as the simpler and more intelligible rules. Modeling was carried out using the parameters shown in Table 4.

Three sets of “IF...THEN” rules were subsequently generated to express the model, one set for each output. IF...THEN rules are made up of two parts: the initial one, which includes the input or inputs explaining a specific output, followed by the second part describing the output characteristics, which are defined by a word and its corresponding membership degree (Table A1) [36].

The predictability of the models was assessed using the determination coefficient (R^2^) defined by Equation (2):

(2) $$R^{2}=\left(1-\frac{\sum_{i=1}^{n}{\left(y_{i}-y_{i}^{\prime }\right)}^{2}}{\sum_{i=1}^{n}{\left(y_{i}-y_{i}^{\prime \prime }\right)}^{2}}\right)\times \;100$$

where y_i_ is the actual point in the data set, y_i_′ is the value calculated by the model and y_i_″ is the mean of the dependent variable. Values of R^2^ must be lower than 99.9%, otherwise there is a risk of overtraining the neural network [50]. The larger the value of the train set R^2^, the more the model captured the variation in the training data. Values for R^2^ > 70% are indicative of reasonable model predictabilities.

The accuracy of the models was evaluated with the analysis of variance to compare predicted and experimental results, respectively. Computed f ratio values higher than critical f values for the degrees of freedom of the model, indicate no statistical significance between predicted and experimental results and hence, model accuracy.

## 4. Conclusions

PGSS^®^ is an advantageous processing technique that allows for the manufacturing of molten substances into solid microparticles, with a special interest for the processing of thermolabile compounds. Melting point measurements of GMS were essential to preliminarily determine the feasible PGSS^®^ operating pressure and temperature conditions. The melting point depletion of GMS lipid under compressed CO_2_ of up to 9 °C is especially relevant from the energy savings and process economics points of view. SLMPs were thus obtained at operating temperatures (57 °C) well below the normal melting point of GMS (61 °C). Artificial intelligence tools combining artificial neural networks and fuzzy logic was as a successful analytical duo to model the production of SLMPs by the PGSS^®^ process. The obtained models served to simplify the understanding of the SLMPs processing through linguistic rules. The model unveiled that the processing pressure and temperature, as well as the nozzle diameter, had a certain influence on the particle size distribution of the SLMPs and yield of particle production. These operating conditions influenced remarkably the mean diameter of the particles, with smaller particles obtained at high temperatures and pressures and small nozzle diameter.

## Figures and Tables

**Table 1:** Yield of particle production, mean diameter and standard deviation of SLMPs of GMS processed using PGSS^®^ technique. Particles were denoted as GMS-x-y-z, where x is the nozzle diameter (mm), y the processing temperature (degrees Celsius) and z the processing pressure (bar).

SLMPs	Mean Diameter (μm)	Standard Deviation (μm)	% Fine Particles
GMS-4-57-120	138.7	47.0	17.4
GMS-4-57-200	182.6	63.3	43.7
GMS-4-62-120	128.0	41.8	12.8
GMS-4-62-200	147.4	48.3	18.3
GMS-4-67-120	103.5	33.1	11.0
GMS-4-67-200	154.3	52.1	27.5
GMS-1-57-120	171.6	56.8	39.5
GMS-1-57-160	172.3	51.6	34.8
GMS-1-57-200	186.2	57.5	25.7
GMS-1-67-120	131.9	44.4	23.5
GMS-1-67-160	130.3	50.0	27.1
GMS-1-67-200	125.4	43.1	34.8

**Table 2:** Inputs selected by FormRules^®^ for the different outputs evaluated in this work, with their respective parameters to evaluate the quality of each model. The most relevant submodels are highlighted in bold.

Output	Submodel	Inputs Selected	R^2^	Degrees of Freedom	f Value	Critical f Value
Mean diameter	1	T	91.5012	5 and 6	12.92	4.39
	2	P × Nozzle				
Standard deviation	1	P × Nozzle	58.3925	4 and 7	2.46	4.12
% fine particles	1	P × Nozzle	75.1098	6 and 5	2.51	4.93
	2	T				

**Table 3:** Nozzle diameters and processing temperatures (T) and pressures (P) tested for the preparation of SLMPs of GMS using the PGSS^®^ technique.

SLMPs	Nozzle (mm)	T (°C)	P (bar)
GMS-4-57-120	4	57	120
GMS-4-57-200	4	57	200
GMS-4-62-120	4	62	120
GMS-4-62-200	4	62	200
GMS-4-67-120	4	67	120
GMS-4-67-200	4	67	200
GMS-1-57-120	1	57	120
GMS-1-57-160	1	57	160
GMS-1-57-200	1	57	200
GMS-1-67-120	1	67	120
GMS-1-67-160	1	67	160
GMS-1-67-200	1	67	200

**Table 4:** Training parameters setting with FormRules^®^ v4.03.

**Minimization parameters**
Ridge Regression Factor: 10^−6^
**Model Selection Criteria**
Minimum Description Length
Number of Set Densities: 2
Set Densities: 2.3
Adapt Nodes: TRUE
Max. Inputs Per SubModel: 2
Max. Nodes Per Input: 10

## Appendix A

**Table A1:** IF…THEN rules generated by FormRules^®^ software. Membership degrees are in parenthesis.

Parameter	Submodel	Rule
Mean diameter	1	IF T is low THEN mean diameter is high (1.0)
		IF T is high THEN mean diameter is low (0.79)
	2	IF P is low and nozzle is large THEN mean diameter is low (1.0)
		IF P is low and nozzle is small THEN mean diameter is high (0.69)
		IF P is high and nozzle is large THEN mean diameter is high (0.69)
		IF P is high and nozzle is small THEN mean diameter is high (0.53)
Standard deviation	1	IF P is low and nozzle is large THEN SD is low (0.63)
		IF P is low and nozzle is small THEN SD is high (0.85)
		IF P is high and nozzle is large THEN SD is high (0.85)
		IF P is high and nozzle is small THEN SD is high (0.78)
% fine particles	1	IF nozzle is large and P is low THEN % particles is low (1.0)
		IF nozzle is large and P is high THEN % particles is high (0.67)
		IF nozzle is small and P is low THEN % particles is high (0.58)
		IF nozzle is small and P is high THEN % particles is high (0.50)
	2	IF T is low THEN % particles is high (0.90)
		IF T is medium THEN % particles is low (0.90)
		IF T is high THEN % particles is low (0.56)
